# Bacterial DNA load in *Staphylococcus aureus* bacteremia is significantly higher in intravascular infections

**DOI:** 10.1371/journal.pone.0266869

**Published:** 2022-04-20

**Authors:** An-Emmie Nieman, Wouter Rozemeijer, Paul H. M. Savelkoul, Rogier P. Schade

**Affiliations:** 1 Department of Medical Microbiology and Infection Control, Amsterdam University Medical Centers, Amsterdam, The Netherlands; 2 Department of Medical Microbiology, School of Nutrition and Translational Research in Metabolism (NUTRIM), Maastricht University, Maastricht, The Netherlands; Universita degli Studi di Parma, ITALY

## Abstract

**Objectives:**

Determination of pathogen-specific bacterial DNA load (BDL) in blood has been shown to be directly correlated with severity of infection in patients with bacteremia. In the diagnostic work-up of patients with *Staphylococcus aureus* bacteremia (SAB), determination of the primary focus is imperative, because of implications for treatment duration, and ultimately prognosis. Here we investigate whether measurement of BDL in patients with SAB can distinguish between intravascular and extravascular foci of infection.

**Methods:**

In a consecutive cohort of 43 patients with positive blood cultures with *Staphylococcus aureus*, we performed a quantitative PCR on whole blood to detect the bacterial DNA load. Infections were classified into 3 categories: i) soft tissue infections and phlebitis, ii) deep-seated infections and iii) endocarditis and other intravascular infections. Bacterial DNA loads and inflammatory parameters in the three categories were analyzed and compared.

**Results:**

Median BDL in patients with endocarditis and other intravascular infections was 1015 cfu/ml, significantly higher than BDL in the other two categories (28 and 31 cfu/ml respectively). In contrast, CRP and leukocytes were not significantly different between the three patient categories. BDL could be detected in all patients with intravascular causes and levels were generally 10–30 times higher than in the other infection categories. Median BDL in non-survivors was 85 cfu/ml, which was higher than in survivors with a median BDL of 29 cfu/ml, although not significant.

**Conclusions:**

In *Staphylococcus aureus* bacteremia pathogen-specific BDL is distinctly higher in patients with intravascular infections compared to extravascular origins. As measurement of BDL by PCR can easily be implemented in routine diagnostics, it can improve the diagnostic work-up of SAB by rapidly identifying the subset of patients who need higher dosages of antibiotics and additional measures to improve outcome.

## Introduction

*Staphylococcus aureus* bacteremia (SAB) is a common and serious infection. It can occur in a wide range of infections, ranging from uncomplicated skin infection or hospital acquired phlebitis to life-threatening conditions such as endocarditis. *Staphylococcus aureus* bacteremia can result in metastatic infections and causes mortality in approximately 30% of patients [1]. Differentiation between uncomplicated and complicated infections is crucial for the choice and duration of therapy. National and international guidelines recommend at least 14 days of intravenous antibiotic therapy, with longer duration in complicated infections [2,3]. Higher doses of antibiotics and intravenous administration during the entire treatment are necessary in endocarditis and intravascular infections and in some cases cardiovascular surgery is required [4,5].

Clinical and microbiological characteristics and radiological imaging can help to identify the presence of a complicated infection. Community acquired infection, persistent bacteremia 72 hours after start of directed therapy and the presence of intravascular prosthetic devices are risk factors for complicated bacteremia and higher mortality [2,6]. One of the most serious *S*. *aureus* infections is endocarditis, which can be difficult to diagnose. The mainstays of diagnosing endocarditis are echocardiography and blood cultures. Adjunctive imaging such as cardiac computed tomographic and nuclear imaging can be used when echocardiography is inconclusive [7]. Specific laboratory parameters for the diagnosis of endocarditis are lacking, therefore, new markers aiding the physician in decision-making are needed. Several studies have suggested that time to blood culture positivity might be used to identify patients with a high likelihood of endovascular infection [8,9]. A short time to positivity is related to a high number of bacteria in the original blood sample, which correlates with an intravascular source of the bacteremia. Quantification of bacterial DNA by real-time polymerase chain reaction (rt-qPCR) is a way to quantify the amount of DNA of a given pathogen directly on whole blood. Observational studies have shown a clear correlation between the level of the bacterial DNA load (BDL) and the severity of infection [10–15].

We performed an observational cohort study in patients with *Staphylococcus aureus* bacteremia to evaluate a putative correlation between the level of pathogen-specific bacterial DNA in blood and the focus of infection.

## Methods

### Patients and sample collection

Over a period of 8 months in 2010/2011, 43 patients having both a positive blood culture with *Staphylococcus aureus* and EDTA blood available were included in the study. Patients were included if EDTA blood was withdrawn within 24 hours from withdrawal of the positive blood culture. EDTA blood was obtained for routine detection of clinical blood parameters, like CRP, hemoglobin, and leucocytes. The remainder of the stored EDTA whole blood was collected at the Laboratory for Clinical Chemistry and Hematology and kept at 4–8°C until DNA isolation.

Clinical strains in blood cultures (BACTEC^TM^ FX, Becton Dickinson) were identified as *Staphylococcus aureus* using the standard laboratory procedures. General patient characteristics and medical diagnosis were registered. To calculate the Pitt bacteremia score [16,17] additional clinical parameters as temperature, hypotension, mechanical ventilation, cardiac arrest and mental status were collected, if available. For patients with the diagnosis endocarditis additional parameters were collected according to the modified Duke criteria [18] as echocardiography results, existence of a new valvular regurgitation and presence of immunological or vascular phenomena. Laboratory parameters—CRP and leukocytes—were registered. These parameters were measured in the same EDTA sample as collected for bacterial DNA load. Normal values of leukocytes were 4,0–10,0*10^9^/l. Normal value of C-reactive protein (CRP) was 0–10 mg/l.

Three categories of infections were made according to severity of disease. First, skin and soft tissue infections (SSTI) including uncomplicated phlebitis, second, deep-seated infections as osteomyelitis, septic arthritis and pneumonia, and third, endocarditis and other intravascular infections, such as infected intracardiac devices.

All diagnoses were based on the clinical judgment of the treating physicians, supported by microbiological and radiographic diagnostics and treated according to relevant national or international guidelines.

This study was overseen by the medical ethics committee of the VU Medical Center. All samples and data were processed anonymously, and the blood samples that were used in the study were all left-overs from diagnostic samples. The medical ethics committee decided to waive the need for informed consent.

### Molecular detection of *Staphylococcus aureus* in blood and measurement of BDL

EDTA blood was kept at 4–8°C and DNA isolation was performed in duplicate within 72 hours. For each DNA isolation, 200μl of EDTA whole blood was used to run protocol A on the NucliSens easyMAG according to the manufacturer’s instructions (bioMérieux, Marcy l’Etoile, France). The eluates (100μl) were frozen to -20°C until amplification. Subsequently, a quantitative real-time PCR was performed on 10μl of eluate, in duplicate, with specific *Staphylococcus aureus* Sa-442 primers described by Martineau et al [19]. All PCR amplifications were performed on a LightCycler 480II (Roche Diagnostics, Pleasanton, USA). Quantification of bacterial DNA loads was calculated based on a standard curve from serial dilutions of a cultured *Staphylococcus aureus* reference strain (ATCC 25923) (resp. 10000; 1000; 100; 10 and 1 cfu/ml equivalents). All PCR amplifications were run with the same serial dilutions of the standard curve. Measurement of BDL was performed in duplicate on each DNA eluate. BDL for each patient was measured by taking the average of all positive results.

### Statistical analysis

Statistical analyses were performed using IBM SPSS® Statistics (version 24). Differences in BDL per patient category were analyzed with the Kruskal-Wallis test and Mann-Whitney U tests. Other markers available (CRP, white blood cell count, Pitt bacteremia score) were also analyzed per patient category using the Kruskal-Wallis test. A Mann-Whitney U test was used for the relation between survival and BDL. A value of p less than 0.05 was considered statistically significant.

## Results

A total of 43 patients were included in the study. The median age was 63 years (range 0–92 years) and 77% of patients was male.

Bacterial DNA on whole blood samples could be detected in 33 of 43 patients (77%) with positive blood cultures with *Staphylococcus aureus*. The level of all bacterial DNA loads ranged from 0 to 5595 CFU/ml.

Twenty-two patients were diagnosed with soft tissue infection or uncomplicated phlebitis. Thirteen patients had deep-seated infections as osteomyelitis, arthritis, pneumonia or urinary tract infection. Eight patients were diagnosed with endocarditis or other intravascular infections.

All *Staphylococcus aureus* strains were methicillin susceptible, except for one MRSA in a patient with endocarditis. Patient characteristics and infection parameters are presented in Table 1, listed by category of infection.

**Table 1:** Patient characteristics and biomarkers.

	Phlebitis & soft tissue infection	Deep-seated infection	Endocarditis & intravascular infection
Number of patients	22	13	8
Male (%)	73%	85%	75%
Age median (yrs) (range)	64 (1–83)	56 (39–78)	68 (0–92)
BDL detectable % patients	68%	77%	100%
BDL median (cfu/ml) (range)	28 (0–421)	31 (0–156)	1015 (86–5595)
Log_10_ BDL median	1,5	1,5	3,0
CRP median (mg/l) (range)	144 (23–336)	233 (64–376)	171 (19–318)
WBC median (*10^9^/l) (range)	12,9 (4,2–31,9)	14,6 (1,5–31,6)	12,9 (5,8–28,1)
Pitt median (range)	1 (0–4) (n = 15)	2 (0–1) (n = 10)	2 (1–4) (n = 7)
In hospital mortality (%)	14%	23%	38%

yrs = years; BDL = bacterial DNA load; cfu = colony forming unit; CRP = C-reactive protein; WBC = white blood cell count.

In the group of patients with endocarditis and intravascular infections, 6 patients were diagnosed with endocarditis, including two with prosthetic valves. Applying the modified Dukes criteria [18], 5 out of 6 patients met the criteria for definite endocarditis and 1 of 6 for possible endocarditis. The other 2 patients in this infection category, had an infected atrial thrombus, and an infected implantable cardioverter defibrillator (ICD) respectively. Bacterial DNA was detected in 8/8 patients (100%) in this category.

The maximum time difference of collection of EDTA blood was 11 hours before, up to 18.5 hours after withdrawal of blood culture, with a mean time difference of 2 hours and 23 minutes and a median of 26 minutes.

**Fig 1:**
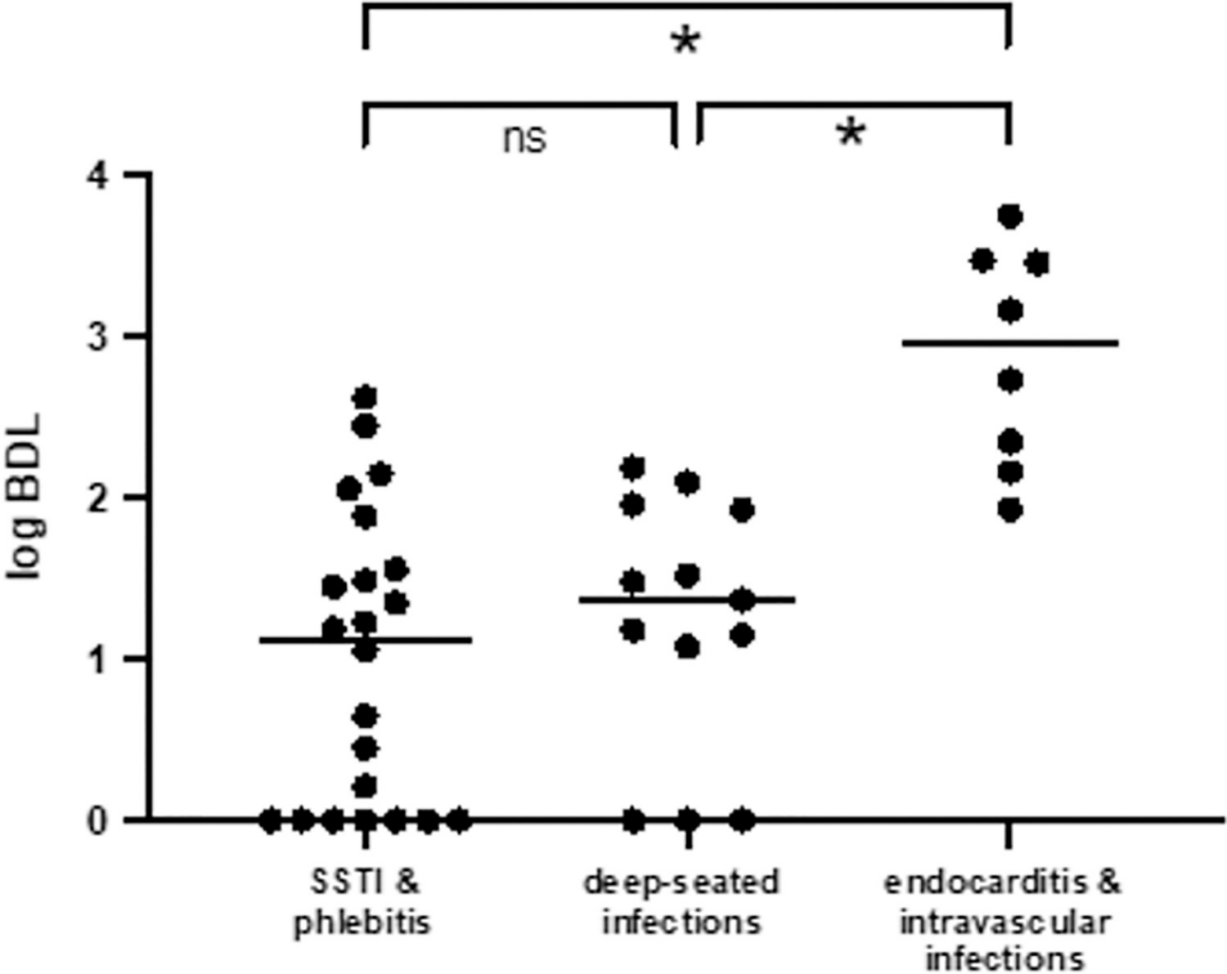
Bacterial DNA loads in three infection categories. SSTI = skin and soft tissue infections; BDL = bacterial DNA load; ns = non-significant difference; inline. = significant difference.

The bacterial DNA load was higher in patients with endocarditis and intravascular infections, compared to the phlebitis/SSTI group, and compared to deep-seated infections. The BDL in the last two infection categories was comparable. Bacterial DNA load was significantly different between the endocarditis group and SSTI group (p = <0.001) and between the endocarditis and deep-seated infections group (p = 0.001) but not between de SSTI group and deep-seated infections group (p = 0.48, Fig 1).

**Fig 2:**
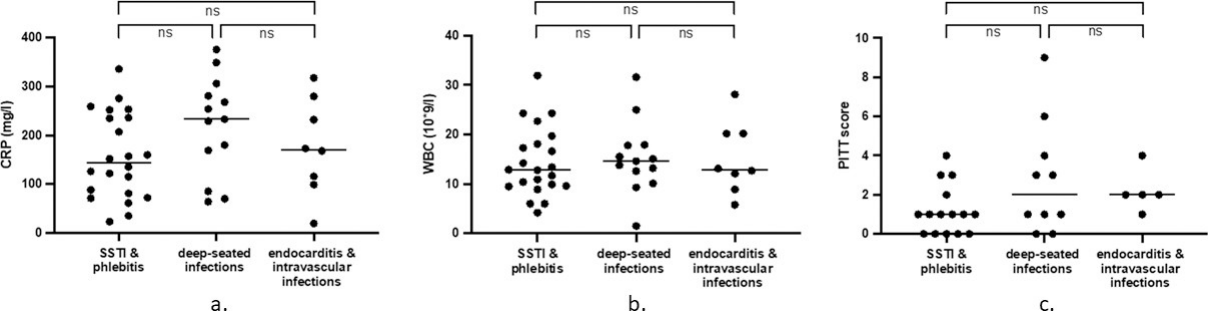
C-reactive protein (CRP) per iection category (A); white blood cell count (WBC) per infection category (B); Pitt bacteremia score per infection category (C). SSTI = skin and soft tissue infections; BDL = bacterial DNA load; ns = non-significant difference.

In contrary to BDL, there was no difference between infection group and CRP (p = 0.20, Fig 2A), white blood cell count (p = 0.80, Fig 2B) or Pitt bacteremia score (p = 0.16, Fig 2C) at the time of first positive blood culture.

Median DNA loads in non-endocarditis infection groups were significantly lower, around 30 cfu/ml, however, a few outliers were found in these groups. None of these patients had DNA loads of 1000 cfu/ml or higher, but there were six patients with a BDL between 100 and 450 cfu/ml. Diagnoses in these patients were wound infection (n = 2), both with persistent fever despite adequate therapy, uncomplicated phlebitis (n = 1), port-a-cath infection with persistent positive blood cultures (n = 1), septic arthritis (n = 1) and osteomyelitis (n = 1).

**Fig 3:**
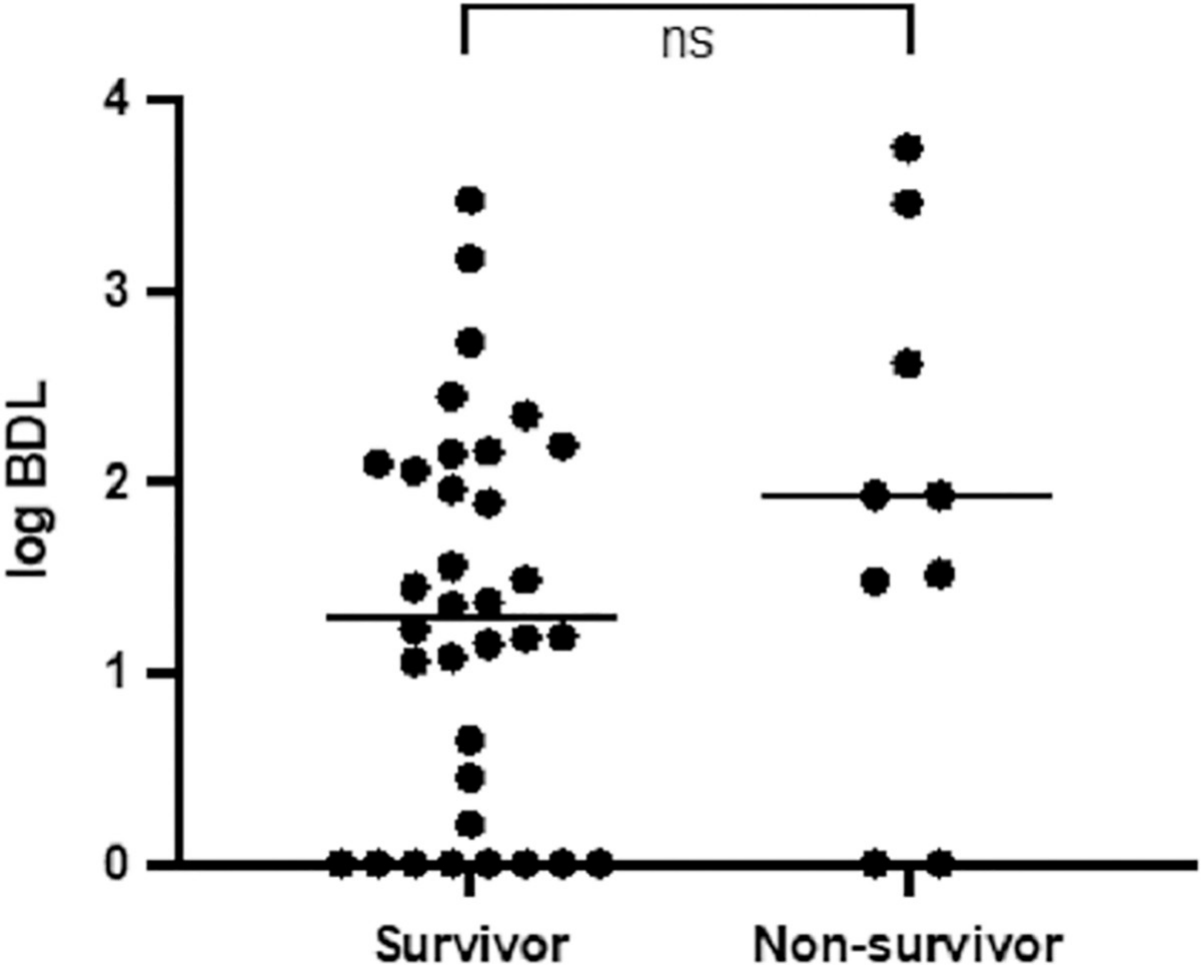
Bacterial DNA load in survivors and non-survivors. SSTI = skin and soft tissue infections; BDL = bacterial DNA load; ns = non-significant difference.

Overall, in hospital mortality was 21%. BDL was higher in non-survivors (Fig 3), but this difference was not significant (p = 0.23).

## Discussion

In this cohort study we measured bacterial DNA loads in blood from patients with positive blood cultures with *Staphylococcus aureus* to investigate whether BDL could be a marker for severity of disease, and infective endocarditis in particular. Patients were divided in three categories according to severity of disease and median BDL between categories was compared. We found that patients with endocarditis and intravascular infections have a significantly higher bacterial DNA load than patients with extravascular origins.

In patients with *S*. *aureus* bacteremia, a specific biomarker for infective endocarditis and other intravascular infections could aid in the decision for dose, duration and route of administration of antibiotic therapy. In our endocarditis and intravascular infection group, all patients had detectable DNA in contrast to the other groups. The median DNA load was 1015 cfu/ml, with a minimum of 86 and maximum of 5595 cfu/ml, compared to a median of around 30 cfu/ml in the other infection categories. The patient with the lowest BDL (86 cfu/ml) in this group, was a patient with a proven prosthetic valve endocarditis, diagnosed with echocardiography. A BDL of 3 log or more was only observed in patients with endocarditis or other intravascular infections. A BDL of 1000 cfu/ml or more was only observed in patients with endocarditis or other intravascular infections. As measurement of BDL by PCR can easily be implemented in routine diagnostics, it can improve the speed of the diagnostic work-up. It is common practice to perform transthoracic echocardiography in patients with *Staphylococcus aureus* bacteremia to screen for endocarditis. However, to rule out intravascular infection, subsequent diagnostic imaging is sometimes needed. If BDL would be included as an additional screening tool for intravascular origin of the SAB, this could lead to more selective use of subsequent transesophageal echocardiography, or nuclear imaging with PET scan. Furthermore, intravascular infections require higher doses of antibiotics, and surgery in some cases. With the addition of BDL as an accessible biomarker, the diagnostic work-up and treatment can be streamlined to improve outcome.

Our results are in accordance with a recent studies [20,21]. Ziegler et al studied 27 patients and demonstrated a relationship between a high *S*. *aureus* DNA load and infective endocarditis and mortality [20]. *S*. *aureus* DNA was measured on blood withdrawn 1 or 2 days after positive blood culture with droplet digital PCR targeting the *nuc* gene. A BDL was detectable in 81% of patients in this study. Guimaraes et al investigated a cohort of 111 patients with complicated *S*. *aureus* bacteremia and measured cell-free *S*. *aureus* DNA [21]. Cell-free bacterial DNA was obtained by using serum that was centrifuged to remove DNA from intact bacteria. The supernatant was used for quantitative PCR. Using this method, the investigators were able to detect DNA in 55% of patients. Patients with infective endocarditis presented with significantly higher DNA compared to other sources of bacteremia, but using cell-free supernatant, not all patients with endocarditis showed detectable DNA levels.

In our study, DNA could be detected in all patients with endocarditis on whole blood. By using whole blood all pathogen-specific bacterial DNA was available for detection, as no cell-free DNA was removed. In addition, blood for PCR was withdrawn around the same time as the blood culture, before start of therapy, compared to blood 1–3 days after the blood culture in the study by Guimaraes.

In intravascular infections, bacteria are present in the blood continuously, which explains the positive results in all patients. In contrast, bacteremia may be intermittent during other infections, which could explain why PCR was negative in some patients with extravascular causes of infection. Of note, BDL results currently have a lower sensitivity compared to blood culture. This is due to the fact that only 10% of 200μl whole blood was used for our amplification compared to 8–10 ml of blood for culture, which is a factor of 400–500 more. Newer techniques that can isolate DNA from larger amounts of blood may therefore further improve the results of BDL measurements [22,23].

The strengths of this study are the efficient selection of patients with *S*. *aureus* bacteremia and the broad patient group, with patients of all ages and types of infection. To measure the most accurate bacterial DNA load we used blood from the same timepoint as the positive blood cultures taken at clinical presentation. Furthermore, we measure pathogen-specific DNA instead of a marker for infection, like C-reactive protein. The level of the bacterial load could aid in the diagnostic decision whether follow-up investigation for endocarditis is needed.

Our study also has limitations. The sample size was limited with a total of 43 patients, and included only 8 patients with infective endocarditis or other intravascular infection. Secondly, we included patients from whom EDTA was available in the Laboratory for Clinical Chemistry that was withdrawn for other reasons. We excluded patients without available EDTA blood and this may have led to selection bias.

Further studies in a large cohort of patients with SAB are needed to control our results. We measured the BDL only at one time point within 24 hours of initial blood culture, but monitoring of BDL at different time points before and during therapy could provide insight into the kinetics of the infection in these patients, and could potentially aid in optimization of therapy.

In conclusion, in our cohort of patients with *Staphylococcus aureus* bacteremia, we found significantly higher bacterial DNA loads in patients with endocarditis and intravascular infections, as compared to patients with extravascular foci. This correlation in non-existent for CRP, leukocytes or Pitt bacteremia score. A DNA load of 1000 cfu/ml or higher was only detected in patients with intravascular infections. These results suggest that the presence of a high BDL can be an indicator for endocarditis and intravascular disease in *S*. *aureus* bloodstream infections. In addition, an undetectable DNA load might rule out endocarditis or intravascular causes of the *S*. *aureus* infection, but this has to be confirmed in a larger cohort.

## Supporting information

S1 Dataset Minimal data set. (PDF)
