# Supplementary material for: Bacterial DNA load in Staphylococcus aureus bacteremia is significantly higher in intravascular infections
Source: PLoS One. 2022 Apr 20;17(4):e0266869. doi: 10.1371/journal.pone.0266869 (PMC9020692; doi:10.1371/journal.pone.0266869)
Supplement: S1 Dataset — (PDF) [file pone.0266869.s001.pdf]

PATIENT CODE	AGE (YEARS)	GENDER (M/F)	BDL MEAN (CFU/ML)	log BDL	CRP0	LEU0	DIAGNOSE	INFECTION CATEGORY	PITT SCORE	IN HOSPITAL MORTALITY (1=DECEASED)
BDL-SA 1	83	M			207	17,3	phlebitis	phlebitis	0	1
BDL-SA 2	67	F	28,05	1,45	157	9,6	wound infection	soft tissue	1	0
BDL-SA 3	69	M	36,7	1,56	35	6	phlebitis	phlebitis	1	0
BDL-SA 4	71	M	1,64	0,21	236	10,4	phlebitis	phlebitis		0
BDL-SA 5	54	M	91,1	1,96	306	17,9	osteomyelitis	deep seated	0	0
BDL-SA 6	74	M	125	2,1	233	10,1	osteomyelitis	deep seated	1	0
BDL-SA 7	45	M	15,1	1,18	254	17,8	geinfecteerde pseudocyste bij pancreas	deep seated		0
BDL-SA 8	22	M			71	12,8	wound infection	soft tissue	0	0
BDL-SA 9	47	M	2,79	0,45	160	31,9	wound infection	soft tissue		0
BDL-SA 10	57	F			61	12,9	wound infection	soft tissue		0
BDL-SA 11	71	M			152	8,9	phlebitis	phlebitis		0
BDL-SA 12	78	M			85	15,5	osteomyelitis	deep seated	3	0
BDL-SA 13	51	M			336	19,7	wound infection	soft tissue		0
BDL-SA 14	64	M			180	13,8	osteomyelitis	deep seated		0
BDL-SA 15	47	M	1490	3,17	280	5,8	endocarditis	endocarditis	2	0
BDL-SA 16	0	F	3030	3,48	19	13,1	meningitis and intravasculair focus CVC	intravasc		0
BDL-SA 17	64	F	11,4	1,06	235	14,2	parotitis	soft tissue	1	0
BDL-SA 18	92	M	540	2,73	318	12,7	endocarditis	endocarditis	1	0
BDL-SA 19	80	F	421	2,62	252	24,3	amputation wound infection	soft tissue		1
BDL-SA 20	39	M	23,6	1,37	268	9,3	osteomyelitis	deep seated	6	0
BDL-SA 21	0,5	M	77,35	1,89	122	18,1	phlebitis	phlebitis		0
BDL-SA 22	68	M	17,05	1,23	126	24,3	wound infection	soft tissue	0	0
BDL-SA 23	0,5	M	224,5	2,35	116	20,2	endocarditis	endocarditis		0
BDL-SA 24	39	F	115,25	2,06	253	22,7	PAC infection	soft tissue	0	0
BDL-SA 25	75	M	22,6	1,35	135	11,7	wound infection	soft tissue	2	0
BDL-SA 26	56	M	29,95	1,48	169	31,6	pneumonia	deep seated	3	1
BDL-SA 27	69	M	5595	3,75	99	28,1	endocarditis	endocarditis	2	1
BDL-SA 28	55	M	30,9	1,49	276	9,9	wound infection	soft tissue	1	0
BDL-SA 29	63	M	4,43	0,65	88	6,0	wound infection	soft tissue	1	0
BDL-SA 30	82	F	15,4	1,19	115	9,5	wound infection	soft tissue	0	0
BDL-SA 31	55	M	14,1	1,15	70	13,2	septische arthritis	deep seated		0
BDL-SA 32	49	F	12,1	1,08	376	12,6	arthritis	deep seated	1	0
BDL-SA 33	74	M	145	2,16	232	20,2	infected ICD	intravasc		0
BDL-SA 34	71	M	0	0	81	4,2	phlebitis	phlebitis	3	1
BDL-SA 35	50	M	155,5	2,19	349	25	septische arthritis	deep seated	0	0
BDL-SA 36	67	V	85,75	1,93	168	8,9	prosthetic valve endocarditis	endocarditis	2	1
BDL-SA 37	63	M	32,9	1,52	281	1,5	UTI	deep seated	9	1
BDL-SA 38	63	M	285	2,45	72	13,4	phlebitis	phlebitis	1	0
BDL-SA 39	81	M	2880	3,46	173	12,1	endocarditis	endocarditis	4	1
BDL-SA 40	60	M	0	0	64	14,6	UTI	deep seated	1	0
BDL-SA 41	50	M	0	0	23	10,9	wound infection	soft tissue (/catheter related)	4	0
BDL-SA 42	62	M	140,5	2,15	259	16,6	wound infection	soft tissue	3	0
BDL-SA 43	75	F	84,85	1,93	229	15,1	osteomyelitis	deep seated	4	1
